# Modulating the Global Response Regulator, LuxO of *V. cholerae* Quorum Sensing System Using a Pyrazine Dicarboxylic Acid Derivative (PDCA^py^): An Antivirulence Approach

**DOI:** 10.3389/fcimb.2017.00441

**Published:** 2017-10-12

**Authors:** M. Hema, Sahana Vasudevan, P. Balamurugan, S. Adline Princy

**Affiliations:** Quorum Sensing Laboratory, Centre for Research in Infectious Diseases, School of Chemical and Biotechnology, SASTRA University, Thanjavur, India

**Keywords:** cholera toxin, LCD, HCD, qRT-PCR, adhesion, invasion

## Abstract

*Vibrio cholerae* is a Gram-negative pathogen which causes acute diarrhoeal disease, cholera by the expression of virulence genes through quorum sensing (QS) mechanism. The QS circuit of *V. cholerae* is controlled by the global quorum regulator, LuxO, which at low cell density (LCD) state produces major virulence factors such as, toxin co-regulated pilus (TCP) and cholera toxin (CT) to mediate infection. On the contrary, at the high cell density (HCD) state the virulent genes are downregulated and the vibrios are detached from the host intestinal epithelial cells, promoted by HapA protease. Hence, targeting the global regulator LuxO would be a promising approach to modulate the QS to curtail *V. cholerae* pathogenesis. In our earlier studies, LuxO targeted ligand, 2,3 pyrazine dicarboxylic acid (PDCA) and its derivatives having desired pharmacophore properties were chemically synthesized and were shown to have biofilm inhibition as well as synergistic activity with the conventionally used antibiotics. In the present study, the QS modulatory effect of the PDCA derivative with pyrrolidine moiety designated as PDCA^py^ against the *V. cholerae* virulence gene expression was analyzed at various growth phases. The data significantly showed a several fold reduction in the expression of the genes, *tcp* and *ct* whereas the expression of *hapR* was upregulated at the LCD state. In addition, PDCA^py^ reduced the adhesion and invasion of the vibrios onto the INT407 intestinal cell lines. Collectively, our data suggest that PDCA^py^ could be a potential QS modulator (QSM) for the antivirulence therapeutic approach.

## Introduction

Quorum Sensing (QS) in bacteria is a system of response and stimuli that depend on the cell density and concentration of autoinducer molecules to co-regulate the biological processes like expression of virulence factors, biofilm, motility, sporulation, bioluminescence etc. (Papenfort and Bassler, 2016; Zhang et al., 2016). Like most of the clinical pathogens, *Vibrio cholerae* which causes severe diarrheal disease, cholera, also uses QS for its virulence mechanism. Hence, manipulating the QS system of *V. cholerae* using target-specific inhibitors/modulators would be a promising anti virulence therapeutic approach, especially in the case of antimicrobial resistance (AMR) strains (Gorski et al., 2016).

In *V. cholera*, the cholera toxin (CT) and toxin-coregulated pilus (TCP) are the major virulence factors which are the under control of QS regulator, LuxO (Zhu et al., 2002). The virulence factor, CT, is encoded by the genes *ctxA* and *ctxB* located in CTXΦ prophage (Maiti et al., 2006). CT is a heterodimeric protein which belongs to an AB_5_ toxin family. It has an enzymatically active single subunit that covalently binds with pentamer B subunit and interacts with GM_1_, a ganglioside receptor. The interaction would translocate the A subunit in an intracellular manner to activate adenylyl cyclase (Polizzotti and Kiick, 2006). The adenylyl cyclase elevates the level of cAMP and alters the ion channels thus effluxing the ions and water (Popoff, 2011).

Colonization of bacteria on the host intestinal epithelial cells is an essential step to establish pathogenesis (Lu and Walker, 2001). The gene, *tcp* in the Vibrio pathogenicity island (VPI) encodes the type IV pilus (TCP). This is co-regulated by CT to mediate the *V. cholerae* intestinal colonization (adhesion) and microcolony formation on the surface of the host cells to promote invasion.

Unlike other pathogenic bacteria, *V. cholerae* expresses its virulence factors at its low cell density (LCD) state. At high cell density (HCD) state, the virulence factors expression is downregulated to enhance the production of the enzyme, protease that detaches the vibrios from the human intestine (Jung et al., 2016). This is facilitated by the transcriptional regulator, LuxO, which acts as a genetic switch between the two distinct modes. The quorum regulator, LuxO, is a member of NtrC type response regulatory protein that purely depends upon ATP hydrolysis as an energy source for its function (Stabb and Visick, 2013). At LCD condition, in the presence of low levels of autoinducers (AIs), the AI receptors act as kinases and transfer a phosphate group to activate the response regulator LuxO. Activated LuxO (LuxO~P) regulates the gene encoding the small regulatory RNAs (sRNAs) Qrr1-4 along with RNA chaperone Hfq binds to the mRNA transcript of HapR (virulence repressor protein in *V. cholerae*) and represses its expression. This further up regulates the gene expression of biofilm and virulence factors including TCP and CT. At HCD state, accumulation of AI leads to the removal of phosphate from LuxO by the phosphatase activity of the AI receptors. The inactive LuxO repress the sRNAs(Qrr1-4) and activates the expression of HapR which further down-regulates the virulence genes expression (Waters et al., 2008; Hema et al., 2015a).

Hence, we propose that targeting LuxO will lead to the premature activation of HCD condition at the LCD state to reduce the level of infection at an early stage (Hema et al., 2015b). Besides, LuxO belongs to NtrC type response regulatory protein and is highly conserved across all *Vibrio* sps., the LuxO selective inhibitors would act as broad spectrum quorum quencher to fight against vibrio infections (Ng et al., 2012). In this context, through our earlier studies, we have shown that 2,3 pyrazine dicarboxylic acid (PDCA) derivatives with pyrrolidine moiety: 3-(4-(Pyrrolidin-1-yl) phenyl carbamoyl) pyrazine-2-carboxylic acid and 3-(3-Fluoro-4-(pyrrolidin-1-yl) phenyl carbamoyl) pyrazine-2- carboxylic acid exhibited anti-biofilm property. In addition, we have also shown that the presence of fluorine group in the latter derivative did not alter the activity of the compound (Hema et al., 2016). Previously, pyrazinamide (a derivative of pyrazine) was shown to have antimycobacterial activity (Mitchison, 1996). It is interesting to note that PDCA was proven to have antibacterial and antifungal properties (Beaula et al., 2015). This suggests that substituted pyrazines might possess antiinfective properties in addition to other biological important activities. Hence, in the present study, the QS modulatory effect of 3-(4-(Pyrrolidin-1-yl) phenyl carbamoyl) pyrazine-2-carboxylic acid termed as PDCA^py^ (Figure S1) was confirmed through gene expression analysis. Further, the host-pathogen relationship was understood through adhesion and invasion studies.

## Materials and methods

### Bacterial strains and culture conditions

*V. cholerae* AMR strain, Vc4, obtained from JSS Medical College, Mysore and reference strain, MTCC 3905, was used for this study. Received strains were cultured in TCBS agar to ascertain cell viability. Pure colonies obtained are preserved in glycerol at −80°C. Strains were grown under standard growth conditions (LB broth, 37°C, aeration) as recommended by the NCCL standard. A LCD of OD_600nm_ = 0.2 was used for further assays (Tyor and Kumari, 2016). For all the assays, PDCA^py^ treatment was carried out at its IC_50_ concentration, 25 μM (Hema et al., 2016).

### RNA extraction and qRT-PCR profiling of gene expression

Total RNA was isolated from *V. cholerae* strains at early-log phase (2 h) and late-log phase (8 h) using RNeasy® Protect Bacteria Mini Kit (Qiagen), according to the manufacturer's guidelines. Integrity and purity of the isolated RNA were checked using standard agarose gel electrophoresis and NanoDrop (Thermo Scientific, USA), respectively. The cDNA was prepared in accordance with manufacturer's instructions of iScript™ cDNA Synthesis Kit. The reaction conditions include annealing at 24°C for 5 min, extension at 42°C for 30 min and the samples were inactivated at 85°C for 5 min.

The expression level of genes under regulation of LuxO was analyzed using qRT- PCR and the primers used for the study were listed in Table 1. The reaction mixture of volume 20 μL contains 1 μL each of forward and reverse primers, 4 μL of diluted cDNA, and 10 μL of the sybr green master mix. The above mixture was made up to 20 μL with RNAse free water. qRT-PCR was performed for 40 cycles as follows: the initial denaturation at 95°C (2 min), denaturation at 95°C (15 s), annealing at 57.7°C (20 s), final extension at 72°C (20 s). Negative control (without cDNA) was maintained in parallel to ensure the samples were free of contamination. The 16 s rRNA was used as reference genes. Relative gene expression was calculated using the 2^−ΔΔCT^ method (Vezzulli et al., 2015).

**Table 1:** Primers used in this study.

**S. no**.	***Gene***	**Primer code**	**Primer sequence (5′−3′)**	**References**
1	*CT*	ct_F	TATGCCAAGAGGACAGAGTGAG	Sarkar et al., 2002
		ct_R	AACATATCCATCATCGTGCCTAAC	
2	*tcp*	tcp_F	CGTTGGCGGTCAGTCTTG	Sarkar et al., 2002
		tcp_R	CGGGCTTTCTTCTTGTTCG	
3	*hapA*	hapA_F	ACGGTACAGTTGCCGAATGG	Silva et al., 2006
		hapA_R	GCTGGCTTTCAATGTCAGGG	
4	*hapR*	hapR_F	CCAACTTCTTGACCGATCAC	Silva and Benitez, 2004
		hapR_R	GGTGGAAACAAACAGTGGCC	
5	*qrr-2*	qrr2_F	GGTGACCCTTGTTAAGCCGA	This study
		qrr2_R	CTATTCACTAACAACGTCAGTTGGC	
6	*qrr-4*	qrr4_F	TGACCCTTCTAAGCCGAGGG	This study
		qrr4_R	GAACAATGGTGTTCACTAACAACG	
7	*16S rRNA*	16sVC_F	ACCTTACCTACTCTTGACATCCA	Lipp et al., 2003
		16sVC_R	CCCAACATTTCACAACACGAG	

### cAMP-assay

Intestinal cell line INT 407 (obtained from NCCS, Pune) was grown in Minimum essential medium (MEM) supplemented with 10% FBS and 1% (v/v) penicillin- streptomycin at 37°C in a 5% CO_2_ atmosphere in T-25 cm^2^ flask. All tissues reagents were purchased from the Himedia Laboratories (Sousa et al., 2001). The cAMP level was quantified using cAMP-Glo™ Promega kit. The assay was performed as per the manufacturer's instructions. In brief, INT 407 cell lines were incubated with CT extracted from the pretreated and control strains. After incubating INT 407 cells for 8 h, the cells were treated with cAMP-Glo™ lysis buffer and incubated for 15 min. Then, 40 μl of cAMP-Glo™ detection solution (2.5 μL Protein Kinase A per 1 mL of cAMP-Glo™ reaction buffer) was added to each well and incubated for 10 min. Following the incubation period, 80 μL of the Kinase-Glo® reagent was added to terminate Protein kinase A (PKA) in all reactions and detect the remaining ATP through luciferase reaction. Further, the plates were shaken for 30 s and incubated for 10 min and a blank was maintained. The luminescence was read with Synergy H1 microplate reader and expressed as RLU which is inversely proportional to cAMP level (Bratz et al., 2013).

### Adhesion and invasion assay

INT 407 cell lines were grown to confluence in MEM medium containing 10% FBS and 1% antibiotics. Cells were grown in 24 well plate and washed three times with sterile cell culture grade PBS and MEM medium (without FBS and antibiotics). In parallel, *V. cholerae* was grown to mid-exponential phase in the presence and absence of the test compound PDCA^py^. The cells were pelleted and resuspended in MEM medium containing 10% FBS without antibiotics. The culture was then added to INT 407 monolayer at a multiplicity of infection (MOI) 50 and incubated for 90 min at 37°C in a 5% CO_2_ atmosphere, for the specific bacterial adherence to the cell monolayer. The non-adherent bacteria were removed by washing the cell lines repeatedly with PBS and MEM containing 10% FBS. After repeated washings, the adherent cells were then detached using 0.1% Triton X-100 in PBS followed by vigorous pipetting. The bacterial CFU was determined by serial plating the diluted suspension. All experiments were performed in triplicates (Chourashi et al., 2016).

The anti-adhesion property of PDCA^py^ further could affect the bacterial invasion to the host cells (Pizarro-Cerda and Cossart, 2006). Hence for invasion assay, in an independent experiment, after infecting the monolayer cell lines with the bacterial culture, the supernatant in each well was replaced with MEM medium containing 10% FBS supplemented with 200 μg/mL gentamycin (to kill adhered extracellular bacteria) and incubated for 60 min at 37°C in a 5% CO_2_ atmosphere. Subsequently, the cell lines were washed three times with PBS and MEM medium. The cell lines were lysed with Triton X-100 and the invaded bacterial cells were counted by plating on LB agar plates (Marini et al., 2015; Peng et al., 2016).

### Microscopic imaging of adherent bacteria using giemsa stain

Sterile coverslips were placed into each well of 24 well tissue culture plates before seeding the cells. After adding the cell suspension over the coverslips, the cells were grown in MEM medium containing 10% FBS and 1% antibiotics for 60 min incubation at 37°C in a 5% CO_2_ atmosphere. After infection, the monolayer was washed with PBS and fixed with methanol for 30 min. Further, the cells were washed with PBS for 3 times and stained for 30 min with Giemsa stain to examine under a microscope (Marini et al., 2015).

### MTT cell viability assay

The cytotoxicity effect of PDCA^py^ was tested on HepG2 cell lines and the cell viability was measured using MTT assay. Hep G2 cells were maintained in Eagle's MEM supplemented with non-essential amino acids, 10% FBS and 1% Pen Strep. Briefly, the cells were resuspended to a density of 1 × 10^6^ CFU/mL and 100 μL were seeded into 96 well plates including a positive control (media and cells without the PDCA^py^) and a blank (media alone). Plates were incubated at 37°C in 5% CO_2_ until the cells reached confluence. Further, the medium was replaced with varying concentration of test compound suspended in MEM medium and incubated for 24 h at 37°C in 5% CO_2._ After the incubation period, 20 μL of MTT solution (5 mg/mL) was added and incubated for 4 h. To this, 100% of DMSO was added to each well, gently swirled for 10 min and the absorbance was read at 570 nm (Kim et al., 2006). The percentage cell viability of PDCA^py^ treated HepG2 cells were calculated comparing with the untreated cells.

### Statistical analysis

GraphPad prism software version 6.05 (GraphPad Software Inc., SanDiego, CA) was used for all statistical analysis. Unpaired *t*-test was used to test the significance. All the assays were conducted in triplicates and the values were expressed as mean ± SD. The value of *P* < 0.05 was used to indicate the significant difference.

## Results

### qRT-PCR profiling of gene expression

The qRT-PCR studies were performed to understand the effect of PDCA^py^ over the expression level of LuxO regulated genes, in clinical isolate (Vc4) as well as in reference strain. The strains without the treatment of PDCA^py^ were taken as control. At LCD state, the LuxO regulated virulence genes *ct, tcp* which encodes for CT and Type-IV regulated co-pilus, respectively, along with small regulatory RNAs (*qrr1-4*) are expressed, repressing the *hapR* and *hapA* which encodes for protease and vice versa in the case of HCD state.

**Figure 1:**
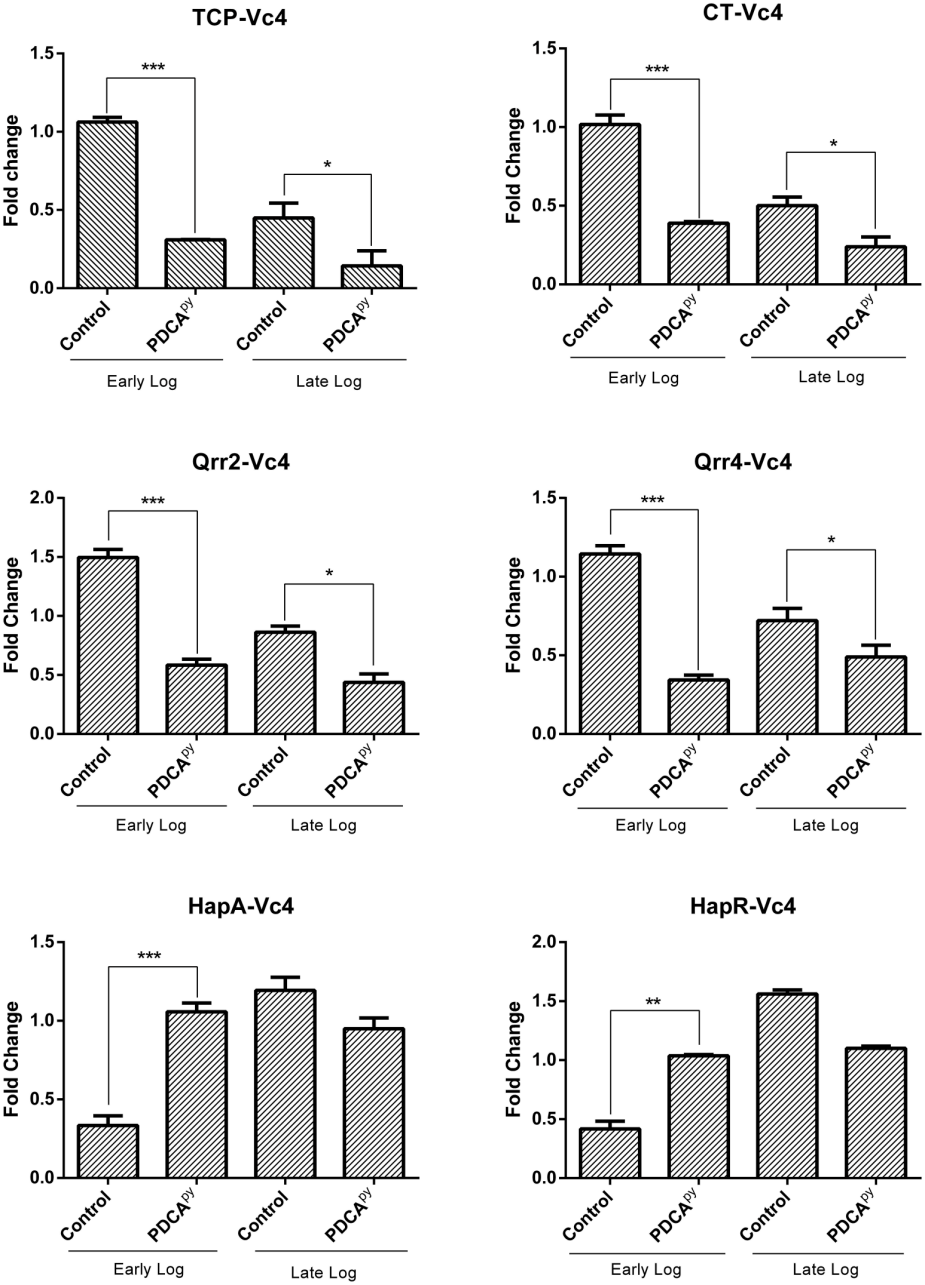
LuxO regulated gene expression analysis by qRT- PCR. The genes *tcp, ct, qrr2*, and *qrr4* showed down-regulation and the up-regulation of *hapR* and *hapA* in the early log phase when treated with PDCA^py^ suggesting premature activation of HCD condition at LCD state. At the late log phase (HCD state), LCD condition is retained in the case of PDCA^py^ treated. 16srRNA was used as reference gene for normalization. Statistical significance denotes, ^***^p ≤ 0.001; ^**^p ≤ 0.01; ^*^p ≤ 0.05.

In our study, PDCA^py^ showed a LuxO selective modulation by significantly down-regulating the expression level of *ct, tcp, qrr-4*, and *qrr-2* at the early log phase. It can be also observed that the *hapR* and *hapA* genes are upregulated at LCD state. A similar trend is sustained at the late log phase (Figure 1). The gene expression of PDCA^py^ treated at the early log phase (LCD state) was comparable to the expression of the control seen at the late-log phase (HCD). This shows the premature activation of HCD condition at LCD state at the genetic level.

### cAMP-assay

**Figure 2:**
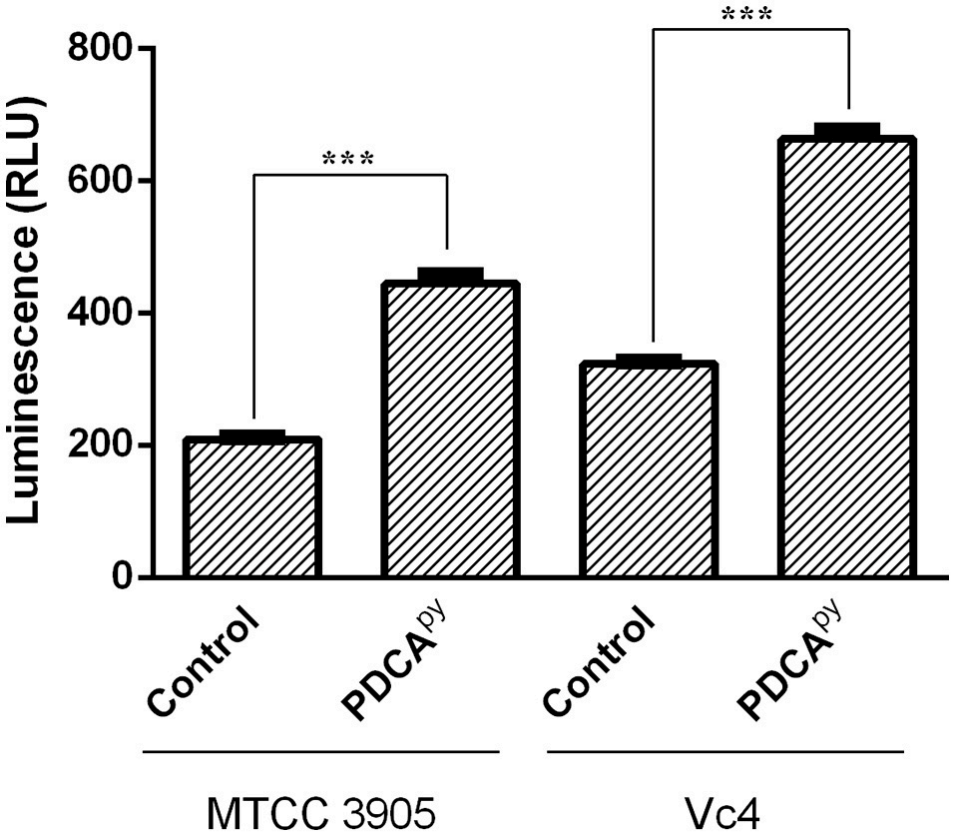
Quorum sensing modulatory effect of PDCA^py^ using cAMP-Glo assay. PDCA^py^ treated samples showed relatively increased luminescence when compared to the untreated samples (control) in both the *V. cholerae* strains, MTCC3905 (reference strain), and Vc4 (clinical isolate).This implies low cAMP levels as it is inversely proportional to luminescence. ^***^Indicates significantly different (*p* ≤ 0.001) compared to untreated control.

The cellular cascade is an ATP-dependent process and the level of ATP and cAMP would directly relate the production of CT as detailed in the introduction (Polizzotti and Kiick, 2006; Popoff, 2011). Figure 2 shows the significant increase in the relative luminescence in the treated (PDCA^py^) cells in comparison with the control. The cell lines infected with either MTCC 3905 or Vc4 on prior treatment with PDCA^py^ showed a relative luminescence expressed as RLU to be 443 and 663, respectively. Also, in the untreated cells, the RLU of MTCC 3905 (control) and Vc4 (control) was found to be 208 and 323, respectively. This implies that the cAMP levels are reduced in the case of treated cells as cAMP levels and RLU are inversely proportional. Thus, the data shows a greater coherence with the gene expression analysis of the LuxO regulated genes by qRT-PCR.

### Adhesion and invasion assay

**Figure 3:**
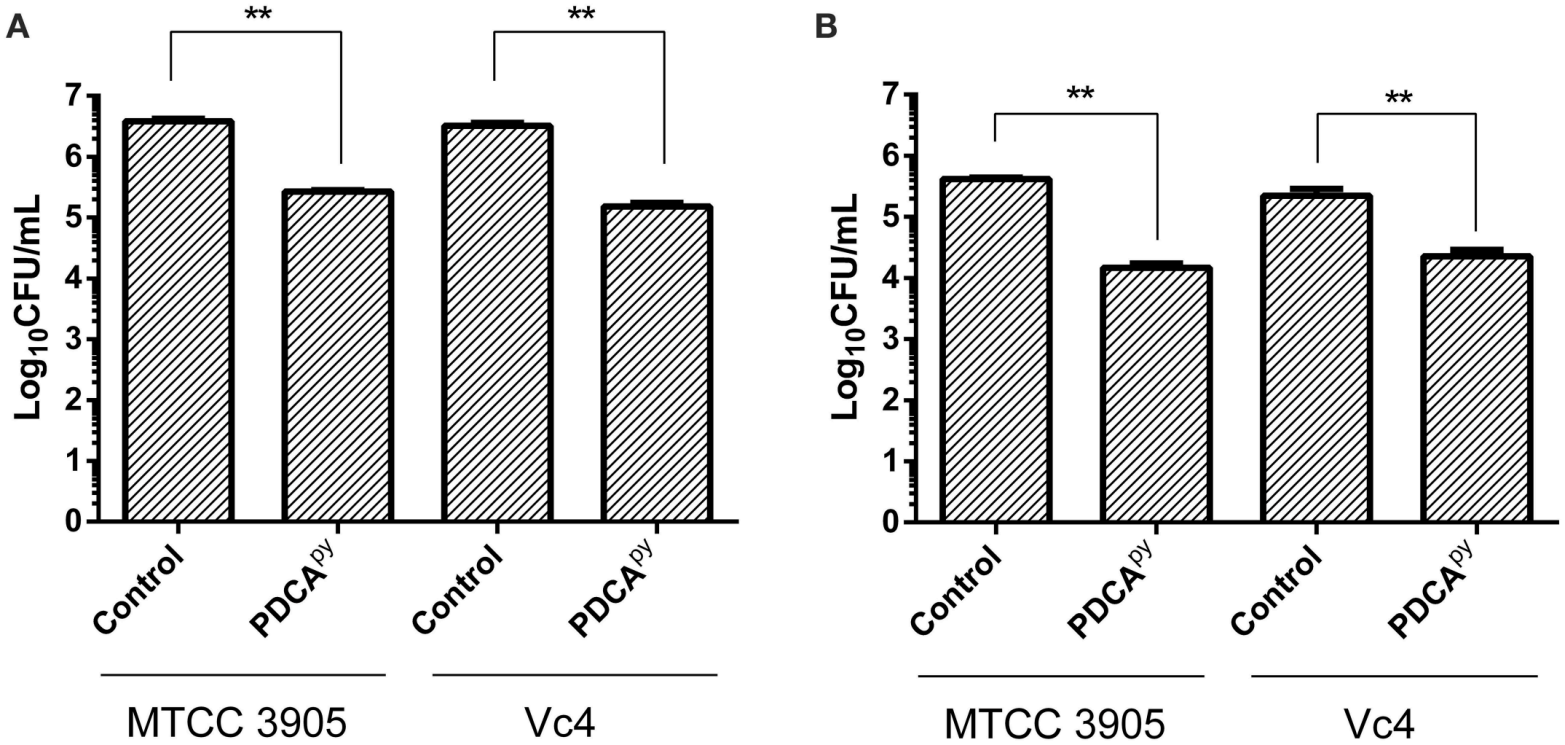
Anti- adhesion and anti- invasion effect of PDCA^py^ treated *V. cholerae* onto INT-407 monolayer cells**. (A)** Adhesion data showing significant reduction in the number of adhered *V. cholerae* onto the intestinal epithelial cells when treated with PDCA^py^. **(B)** Invasion results where the number of invaded cells enumerated was significantly reduced in the case of PDCA^py^ treated bacterial cells. ^**^Indicates significantly different (*p* ≤ 0.01) compared to untreated control.

From qPCR expression profiling, it was elucidated that the gene *tcp* (encoding Toxin Co-regulated Pilus; TCP) expression was down-regulated in the presence of PDCA^py^ and so we intended to examine its effect on the adhesion of *V. cholerae* to INT 407 cell monolayers. In the case of Vc4, the anti-adhesion effect of PDCA^py^ showed a significant reduction of 1.53 × 10^5^ CFU/mL when compared to the untreated cells (3.23 × 10^6^ CFU/mL). Similar results were observed for MTCC 3905 (Figure 3A). In invasion assay (Figure 3B), the treated cells with PDCA^py^ showed a greater reduction in the invaded cells (Vc4) of about 2.2 × 10^4^ CFU/mL when compared to the untreated control (2.2 × 10^5^ CFU/mL). Similar observations were made for MTCC 3905 (treated: 1.4 × 10^4^CFU/mL, untreated: 4.1 × 10^5^ CFU/mL). The data were in concordance with the qRT-PCR expression profile of LuxO regulated genes, where the TCP expression level was down-regulated by the quorum modulators, PDCA^py^ that invariably showed its effect in the bacterial adherence to the INT 407 cell lines.

**Figure 4:**
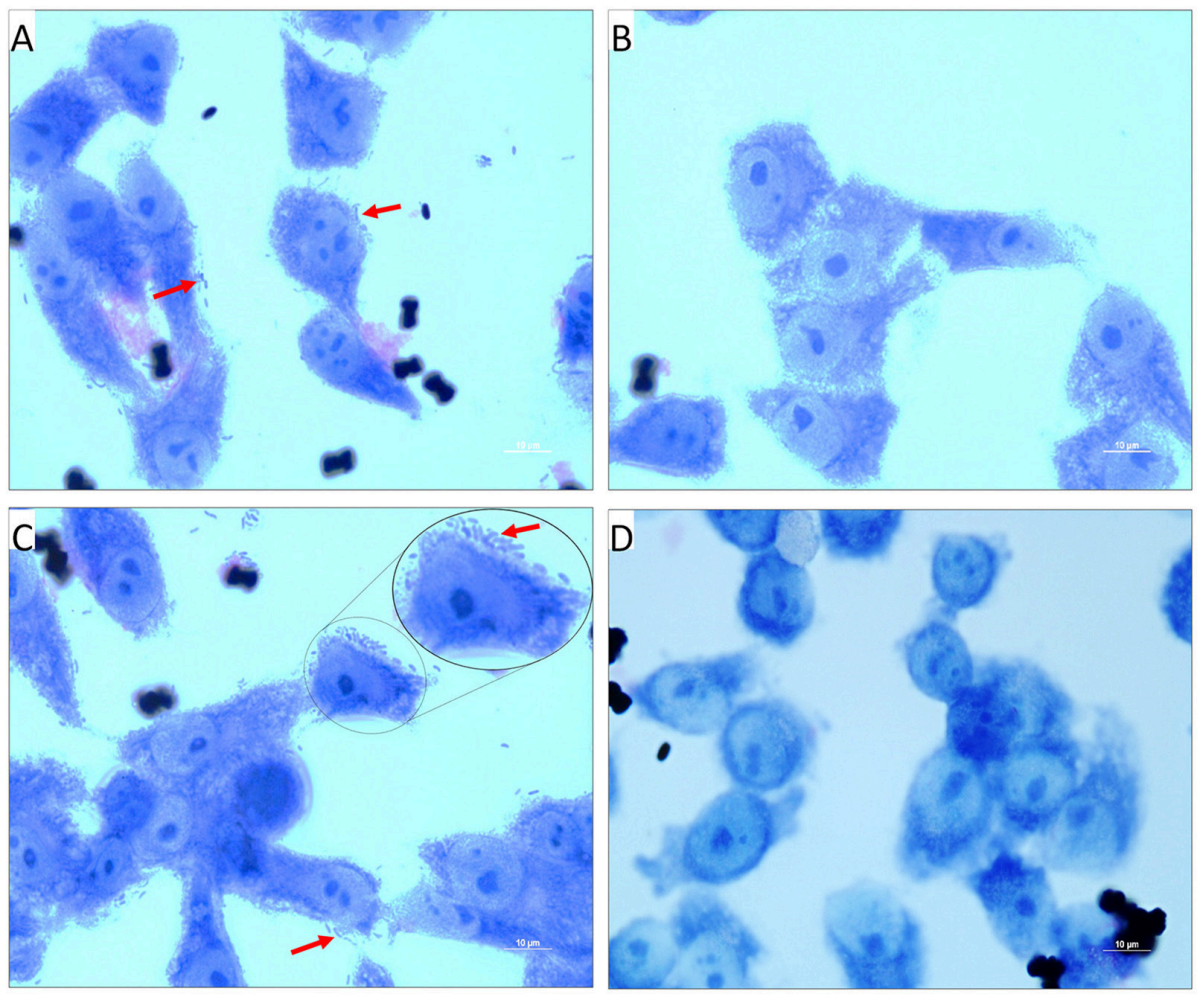
Light micrographs showing the adhered *V. cholerae* onto INT 407 monolayer cells. **(A,C)** Control panels of INT 407 cell lines infected with MTCC3905 reference strain and clinical isolate, Vc4, respectively. The red arrow mark represents the adhered bacterial cells onto the intestinal cell lines producing a carpet like aggregate pattern. **(B,D)** PDCA^py^ treated MTCC3905 reference strain and clinical isolate, Vc4 bacterial cells adhered to INT 407 cells, respectively. PDCA^py^ treated cells, showed a significant reduction in adherence to the cell lines as compared to the control. Scale bar in micrographs represents 10 μm.

**Figure 5:**
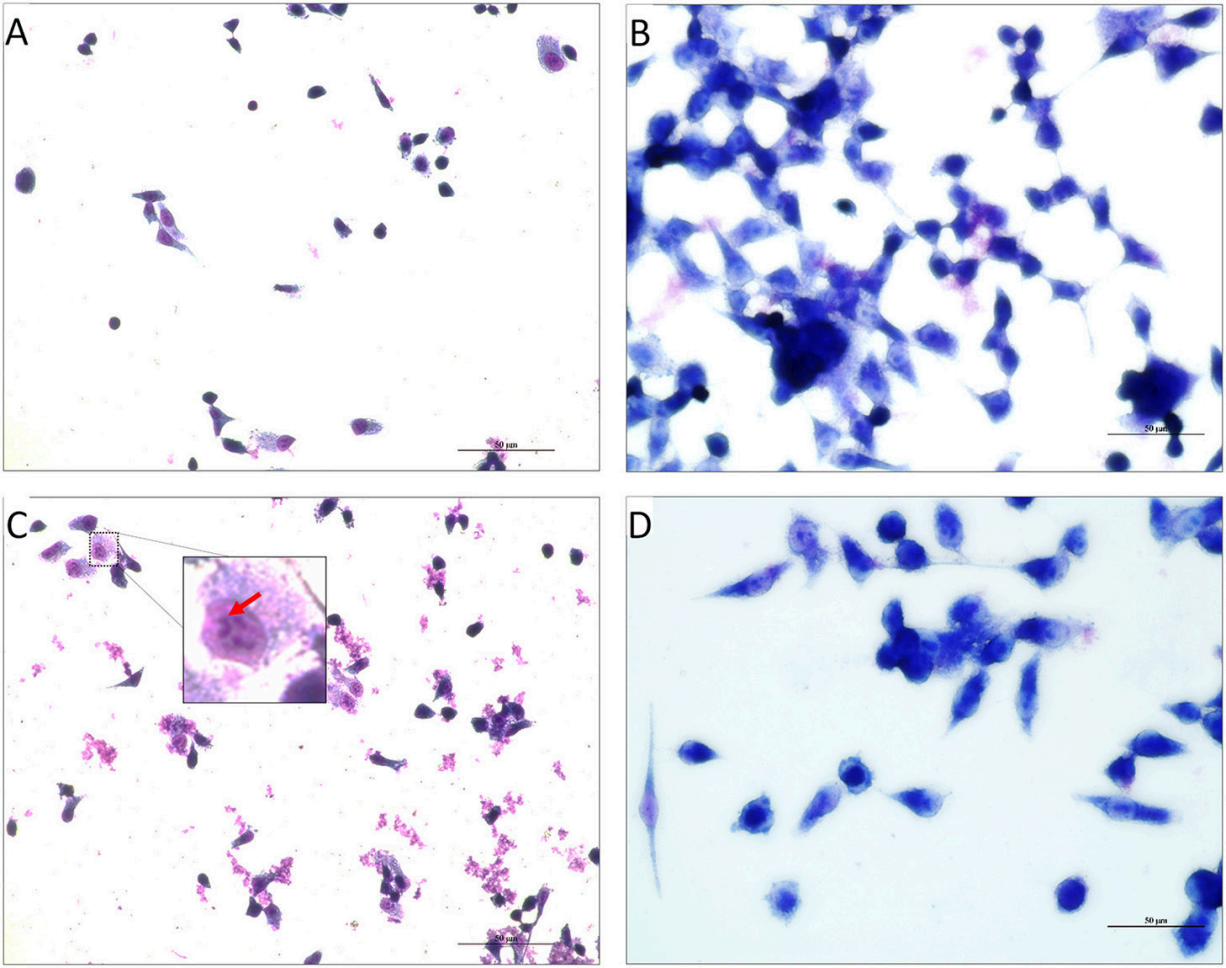
Light Micrographs showing the invaded *V. cholerae* onto INT 407 monolayer cells. **(A,C)** INT 407 cell lines invaded by the PDCA^py^ untreated MTCC3905 and Vc4, respectively. The coma shaped *V. cholerae* cells that are invaded in the INT 407 cell lines can be distinctly seen in the untreated control (inset, enlarged image).The invasion of the *V. cholerae* cells into intestinal epithelial cells were unhealthy (rounded and detached). **(B,D)** PDCA^py^ treated *V. cholerae* showed reduced invasive property, leading to healthy intestinal cells. Scale bar in micrographs represents 50 μm.

Further, TCP-mediated cell adherence of *V. cholerae* onto the INT 407 cell lines in the presence and absence of PDCA^py^ was visualized by light microscope. The light micrographs of adhesion assay (Figure 4) shows that the untreated bacterial cells (comma shaped *V. cholerae* cells) are adhered onto the INT 407 cell lines. On the contrary the PDCA^py^ treated cells showed significantly reduced adherence substantiating the CFU reduction (Figure 3A) in the PDCA^py^ treated bacterial cells. Similarly, the invasion assay showed detachment of INT 407 cells which illustrates the invasion of the PDCA^py^ untreated bacterial cells. In the case of treated bacterial cells, invasion is reduced thereby maintaining the intact monolayer of INT 407 cell lines (Figure 5).

### MTT cell viability assay

**Figure 6:**
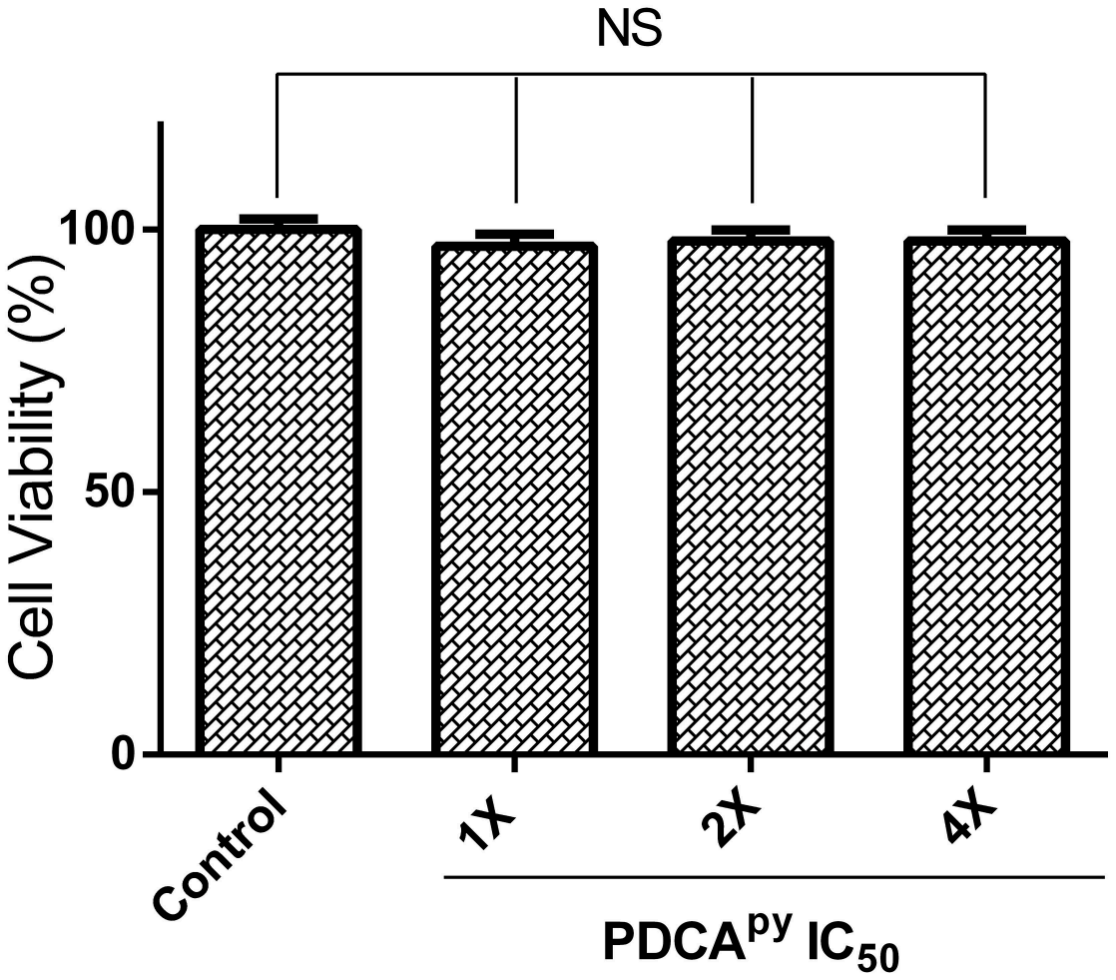
Cell viability of HepG2 cells treated with PDCA^py^. Cell viability of HepG2 cells was not affected as indicated by the insignificant difference between control and treated at its IC_50_ concentration, 1X = 25 μM, 2X = 50 μM, 4X = 100 μM. NS denotes no significant differences (*p* > 0.05).

Cell-based MTT assay was used to investigate the toxic effect of a synthesized compound, PDCA^py^ on HepG2 cell line (Soldatow et al., 2013). The reduction of yellow tetrazolium MTT (3-(4,5-dimethylthiazolyl-2)-2,5-diphenyltetrazolium bromide) is reduced in metabolically active cells as a part of an action of dehydrogenase enzymes, to generate reducing equivalents (NADH, NADPH). The results (Figure 6) showed that the percentage viability of the HepG2 cell line was unaffected in the presence of the test compound at its 2-fold and 4-fold IC_50_ concentrations compared to the untreated control cells.

## Discussion

The intestinal pathogen *V. cholerae*, encodes virulence factors like TCP, CT, and biofilm for their persistence in the host cells leading to acute diarrhoeal disease, cholera (Reidl and Klose, 2002). As QS plays a crucial role in pathogenic vibrio to regulate biofilm formation and virulence factors, chemical or natural molecules that could modulate the QS system could be a rational alternative therapy (Ng et al., 2012; Faloon et al., 2014). LuxO is the global response regulator for the known QS signaling pathways in *V. cholerae* (Cheng et al., 2015). Previous reports also state that LuxO targeted pro-quorum sensing molecules which lock the pathogenic vibrios into the HCD QS mode at the LCD state could be exploited as the aforementioned therapeutic approach (Ng et al., 2012). The premature activation of HCD condition at LCD state will prevent the biofilm formation and the expression of virulence factors promoting detachment of vibrios from the intestine. Such modulators will facilitate the clearance of detached vibrios by the host immune response and prevent the overuse of antibiotics by their synergistic action with the conventional antibiotics (Hema et al., 2016). In addition, these modulators do not pose a survival stress to the bacteria, thus minimizing the resistance development. Hence, targeting LuxO would be a promising anti-virulence therapeutic strategy.

In the light of our previous *in silico* studies, we had shown that target specific modulators, PDCA and its derivatives, could impede the activity of LuxO as they interact with three key amino acids (G170, G172, and I140) present in the ATPase domain which hydrolyzes ATP molecules (Hema et al., 2015b). Further, the synthesized pyrrolidine derivative of PDCA was proven to possess anti-biofilm activity without affecting the growth of *V. cholerae* (Figure S2) speculating to the LuxO targeted QS modulatory effect (Hema et al., 2016). Hence, the present work is to explore the mechanism underpinning the LuxO modulatory activity by 3-(4-(Pyrrolidin-1-yl) phenyl carbamoyl) pyrazine-2-carboxylic acid (PDCA^py^) thus providing the prelude for probing PDCA-based novel quorum-sensing modulators.

Gene expression profiling provides an insight to elucidate the pathway that could be altered by the QS modulators (QSMs). In *V. cholerae*, the infection cycle initiates at LCD state with the up-regulated virulence gene expressions like *ct, tcp*, regulatory RNAs *qrr1-4* and down-regulated *hapR* gene expression, thus, promoting intestinal colonization. Subsequently at HCD state, the dissemination of colonized vibrios from the human intestinal cells is facilitated with the upregulation of *hapR* and down-regulation of the other virulence genes (Ng et al., 2012).

In the present study, down-regulated gene expression of *ct, tcp, qrr*, and up-regulated gene expression of *hapA, hapR* is reported at the early-log phase for the PDCA^py^ treated cells (Figure 1). The data showed that PDCA^py^ modulates the QS genetic circuit to mimic the gene expressions of HCD condition to prevail at the LCD state. This further inhibits the phenotypic expression of CT and TCP virulence factors. In a similar study the effect of thio-azauracil based broad spectrum pro-quorum sensing molecules on down-regulation of virulence gene expression in *V. cholerae* was investigated (Ng et al., 2012).

The release of CT initiates the synthesis of cAMP by activating the adenylyl cyclase to regulate the cystic fibrosis transmembrane conductance regulator (CFTR). This leads to an instant efflux of ions and water from the infected intestinal cells to cause diarrhea (Gurney et al., 2017). Literature reports suggest that cAMP is an indirect measure to relate the expression of CT (Hyun and Kimmich, 1982; Bratz et al., 2013). Hence, here we have used cAMP-Glo assay to determine the cAMP levels in PDCA^py^ treated and untreated conditions. According to this assay, cAMP binds to protein kinase A to release the active catalytic subunits. This further catalyzes the transfer of the terminal phosphate of ATP to a protein kinase A substrate, consuming ATP in the process. Thus, the level of remaining ATP is measured in terms of luminescence which is inversely proportional to cAMP levels (Bratz et al., 2013). Here we have observed increased luminescence in the PDCA^py^ treated cells as compared to the control, indicating the decreased levels of CT. This is reinforced by the evidence from gene expression studies with significant down-regulation of *ct* (Figure 1).

The colonization factor, type IV TCP, enhances *V. cholerae* pathogenesis promoting the formation of micro-colonies in the intestine (Millet et al., 2014). Almagro-Moreno et al. (2015) had demonstrated that Δ*tcp* strains neither colonize the human epithelial cells nor cause the key symptoms of cholera. Hence, the anti-adhesion effect of PDCA^py^ was demonstrated in the human intestinal epithelial cell line, INT 407. The reduced CFU counts from the PDCA^py^ treated bacterial cells suggest the interference in adhesion to INT 407 cell lines. This is attributed to the fact that PDCA^py^ specifically modulated the LuxO and subsequently the expression of TCP, a major colonization factor. The property of invasion can be considered as another aspect of *V. cholerae* pathogenicity, similar to enterotoxigenic *Escherichia coli* infection (Elsinghorst and Kopecko, 1992). Similar to the adhesion assay, a significant reduction in the invasion was also observed. This is corroborated from the light microscopic images (Figures 4, 5) that PDCA^py^ interferes with the colonization onto the intestinal cells. These results are in concordance with the reduced gene expression of the colonization factor, *tcp* (Figure 1).

For a small molecule to be scored as a drug candidate, one of the essential characteristics is non-toxicity toward the host cell. In this context, MTT-based cytotoxicity studies have shown the non-toxic nature of the QSM, PDCA^py^. Additionally, the other drug-like properties that include water solubility, cell permeability, good absorption and no host cell cytotoxicity as reported earlier through our *in silico* studies (Hema et al., 2015b).

Targeting the ATPase domain of LuxO could potentially be developed as broad spectrum NtrC family inhibitors as it is highly conserved in all *Vibrio* sp. (Boyaci et al., 2016). Ng et al. (2012) have tested their LuxO targeted pro-quorum sensing molecules of *V. cholerae* against *V. harveyi* and *Vibrio parahaemolyticus*. They have concluded that these molecules are capable of modulating QS in other *Vibrio* sp. also where LuxO functions to be the global QS regulator (Ng et al., 2012). In this context, PDCA^py^ could serve as a potent broad spectrum lead molecule for *Vibrio* sp. infections.

## Conclusion

QS is a well-known system that regulates biofilm and various virulence factors in pathogenic bacteria. Our studies provide an insight on the QS modulatory effect of PDCA^py^ [3-(4-(Pyrrolidin-1-yl) phenyl carbamoyl) pyrazine-2-carboxylic acid] against both clinical as well as reference strains of *V. cholerae*. Thus, PDCA^py^ could serve as a potent, target-specific and non-toxic lead for drug development against Vibrio infections.

## Author contributions

All authors listed, have made a substantial, direct and intellectual contribution to the work, and approved it for publication.

### Conflict of interest statement

The authors declare that the research was conducted in the absence of any commercial or financial relationships that could be construed as a potential conflict of interest. The reviewer YGT and handling Editor declared their shared affiliation.

## Supplementary material

Figure S1 Chemical Structure of PDCA^py^ (3-(4-(Pyrrolidin-1-yl) phenyl carbamoyl) pyrazine-2-carboxylic acid).

Figure S2 Growth curve of *V. cholerae* MTCC 3905 and Vc4 strains in the presence and absence of PDCA^py^ at its IC_50_ Concentration, 25 μM. The growth of the *V. cholerae* strains taken was not affected in the presence of PDCA^py^.
