# Supplementary material for: Modulating the Global Response Regulator, LuxO of V. cholerae Quorum Sensing System Using a Pyrazine Dicarboxylic Acid Derivative (PDCApy): An Antivirulence Approach
Source: Front Cell Infect Microbiol. 2017 Oct 12;7:441. doi: 10.3389/fcimb.2017.00441 (PMC5643417; doi:10.3389/fcimb.2017.00441)
Supplement: Figure S1 — Chemical Structure of PDCApy (3-(4-(Pyrrolidin-1-yl) phenyl carbamoyl) pyrazine-2-carboxylic acid). [file DataSheet1.DOCX]

**Modulating the global response regulator, LuxO of *V. cholerae* quorum sensing system using a pyrazine dicarboxylic acid derivative (PDCA^py^): An antivirulence approach**

M. Hema, Sahana Vasudevan, Balamurugan P and S. Adline Princy^*^


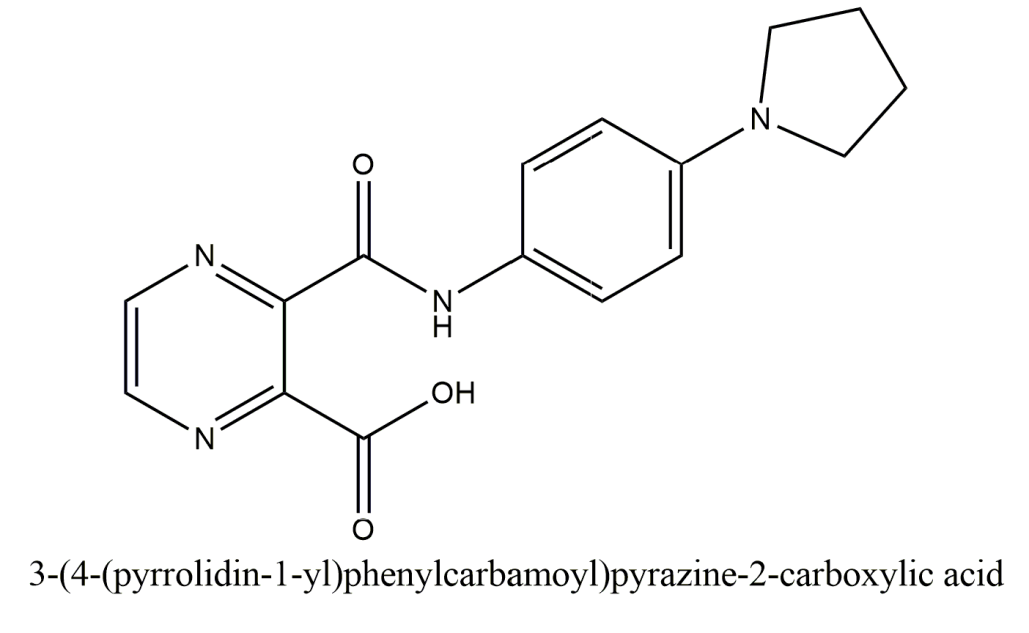


**Figure S1 Chemical Structure of PDCApy (3-(4-(Pyrrolidin-1-yl) phenyl carbamoyl) pyrazine-2-carboxylic acid)**

**

**

**Figure S2 Growth curve of *V. cholerae* MTCC 3905 and Vc4 strains in the presence and absence of PDCA^py^ at its IC_50_ Concentration, 25 µM.** The growth of the *V. cholerae* strains taken was not affected in the presence of PDCA^py^.
